# A Case of Amenorrhœa

**Published:** 1849-02

**Authors:** James R. Wilcox

**Affiliations:** Mile Grove, Indiana


ART. V.—*A Case of Amenorrhœa.* By JAMES R. WILCOX, M.D., of Mile Grove, Indiana.

Caroline W., the subject of this case, is about 19 years of age, of rather feeble constitution, and unmarried. Her health, previous to the occurrence of amenorrhœa, had always been excellent, with the exception of occasional attacks of intermittent fever. Sometime in the month of February, 1847, she did a hard days work, washing, during the time of her catamenial discharge, and from that time on discovered an entire suppression of the menses. In about six months I was called to visit her. I found her in bed, with her hands upon her head, complaining of nothing but blindness and a severe headache. I made some inquiries into the case and could learn nothing concerning it more than she had for some time complained of headache, and was suddenly taken blind, and that they had noticed her for some months looking rather paler and less healthy than usual. I examined her pulse and found it somewhat excited, full, and strong. I also examined the eyes and found the pupils considerably dilated, and not at all influenced in their movements by the light of a candle. I immediately bled her to the amount of ten to twenty ounces. Before the blood had ceased running she was almost entirely relieved of headache, and her sight was also somewhat restored, and in the course of eight hours was as good as ever. I then ordered a dose of calomel, colocynth, and jalap. The medicine operated copiously in the course of six or eight hours. In a few days, without further treatment, she was able to attend to business, though very feeble.

On inquiring particularly of her mother, I learned the state of her menstrual function, and prescribed accordingly. The usual emmenagogues were given, and exercise on horseback recommended, but to no purpose, her health continuing to grow worse. Once a month, about the time when the menses should appear, she experienced a recurrence of the blindness. She was almost immediately relieved by relieving the bloodvessels of the brain, by either general or local depletion, but still the case progressed, and on the 25th of September, about seven months from the time of the suppression, at one of her monthly periods, in addition to the blindness and other symptoms that had attended, she was seized with convulsions, which lasted for about forty-eight hours. After this she began to improve, and under the use of emmenagogues, aided by suitable exercise, is fast regaining her health.

December, 1848.

